# Standardizing digital biobanks: integrating imaging, genomic, and clinical data for precision medicine

**DOI:** 10.1186/s12967-024-04891-8

**Published:** 2024-02-05

**Authors:** Valentina Brancato, Giuseppina Esposito, Luigi Coppola, Carlo Cavaliere, Peppino Mirabelli, Camilla Scapicchio, Rita Borgheresi, Emanuele Neri, Marco Salvatore, Marco Aiello

**Affiliations:** 1 IRCCS SYNLAB SDN, 80143 Naples, Italy; 2 Bio Check Up S.R.L, 80121 Naples, Italy; 3 Department of Advanced Biomedical Sciences, University of Naples Federico II, 80131 Naples, Italy; 4 UOS Laboratori di Ricerca e Biobanca, AORN Santobono-Pausilipon, Via Teresa Ravaschieri, 8, 80122 Naples, Italy; 5 Academic Radiology, Department of Translational Research, University of Pisa, via Roma, 67, 56126 Pisa, Italy

**Keywords:** Big data, Biobanking, Standardization, Data integration, Imaging, NGS, Clinical decision support systems (CDSS), Radiomics, Pathomics, Precision medicine

## Abstract

Advancements in data acquisition and computational methods are generating a large amount of heterogeneous biomedical data from diagnostic domains such as clinical imaging, pathology, and next-generation sequencing (NGS), which help characterize individual differences in patients. However, this information needs to be available and suitable to promote and support scientific research and technological development, supporting the effective adoption of the precision medicine approach in clinical practice. Digital biobanks can catalyze this process, facilitating the sharing of curated and standardized imaging data, clinical, pathological and molecular data, crucial to enable the development of a comprehensive and personalized data-driven diagnostic approach in disease management and fostering the development of computational predictive models. This work aims to frame this perspective, first by evaluating the state of standardization of individual diagnostic domains and then by identifying challenges and proposing a possible solution towards an integrative approach that can guarantee the suitability of information that can be shared through a digital biobank. Our analysis of the state of the art shows the presence and use of reference standards in biobanks and, generally, digital repositories for each specific domain. Despite this, standardization to guarantee the integration and reproducibility of the numerical descriptors generated by each domain, e.g. radiomic, pathomic and -omic features, is still an open challenge. Based on specific use cases and scenarios, an integration model, based on the JSON format, is proposed that can help address this problem. Ultimately, this work shows how, with specific standardization and promotion efforts, the digital biobank model can become an enabling technology for the comprehensive study of diseases and the effective development of data-driven technologies at the service of precision medicine.

## Introduction

Within the healthcare system, the rapid development of modern diagnostic techniques has resulted in an explosion of data production, much of which is heterogeneous and belongs to different domains. Indeed, innovations in imaging technologies, such as MRI, CT, PET scans, and advanced microscopy, provide detailed anatomical and functional information at various scales. Next-generation sequencing (NGS) technologies have revolutionized the acquisition of genomic data. NGS are high-throughput methods that allow for rapid and cost-effective sequencing of entire genomes, exomes, or specific gene panels. This wealth of genetic information enables identification of genetic variants associated with diseases, drug responses, and personalized treatment strategies. This knowledge drives the development of targeted therapies, where drugs are tailored to an individual’s genetic makeup, optimizing treatment efficacy and minimizing adverse effects. Clinical imaging, pathology and NGS represent the most advanced sectors, bringing the most significant amount of information for clinical research and decision-making across various medical fields, including cardiology, neurology, infectious diseases and, in particular, oncology [1–5]. This information can offer a complete view of the complex biological phenomena at different scales (such as histological and clinical imaging) and different characteristics (genotypic and phenotypic) of diseases, making it valuable to release curated raw data [6–8].

The concept of biobank precisely addresses this need. In fact, the guiding principles of the “Biobank Act” are the promotion of trustworthy, equal access to data and samples, protection of privacy, acceleration of innovation activities and exposing biobank activities to public scrutiny [9].

The aim of the biobanking is to ensure the availability of qualitatively annotated biological samples for planning research programs, and to foster innovative and personalized approach to disease treatment and diagnosis [10]. The more well-characterized, high-quality samples and associated data are available through biobanks, the faster research will progress and impact today’s healthcare.

As biobanks are important sources for the provision of research-ready samples as well as associated data, they can face a dual bottleneck of data harmonization and curation [11]. These aspects are interconnected, and both can directly affect the biobank suitability.

Variations associated with collecting, processing, and storing procedures make it extremely hard to extrapolate or to merge data from different domains (i.e., imaging, pathology, and molecular profiling) or institutions. For instance, if one institution uses a different coding system for diagnoses compared to another, merging this data without proper harmonization could lead to erroneous conclusions or missed insights. Another example: genomic research often involves data collected from various laboratories using different techniques and platforms. Harmonizing genetic data requires aligning genetic markers, normalizing gene expression values, and reconciling discrepancies in annotations. Without proper harmonization, comparing data across studies becomes unreliable, impacting the accuracy of genetic associations or findings. Inconsistencies in data collection or storage methodologies could compromise the validity of longitudinal studies investigating disease progression or treatment outcomes. It is easy to introduce invisible bias, leading to irreproducible findings. Therefore, the standardization and harmonization of biobanking practices are of paramount importance [11]. Another fundamental aspect to be explored is the generation of numerical descriptors associated with each single domain. This process passes through the definition of robust data curation and processing procedures.

Dealing with standardized and harmonized procedures is a fertile ground for both developing omics studies (e.g., radiomics, pathomics, genomics), as well as for the exploration of the potential links between -omics quantitative data and clinical outcomes of patients with specific diseases, primarily cancer [3]. In particular, the challenges associated with integrating data from diverse domains are multifaceted and can significantly impact the overall quality and interpretability of integrated datasets. One major obstacle stems from the heterogeneity in data formats across these domains, as each field often adopts distinct formats. Additionally, biological variability, inherent in living systems, manifests differently across domains. Therefore, integrating data across-domains requires careful consideration of biological variations to ensure that observed patterns are genuine and not artifacts of the integration process. Furthermore, differences in data resolutions present another hurdle. While imaging data might possess high spatial resolution, molecular data may operate at the molecular or genomic level. Integrating datasets with varying resolutions necessitates meticulous consideration to prevent loss of information or misinterpretation during the integration process. All these challenges presuppose having high-quality numerical descriptors for each domain.

These considerations highlight the need to put together different diagnostic and clinical domains in a comprehensive manner through a digital approach, while promoting data sharing and biobank sustainability. The concept of digital biobank, namely ecosystem of readily accessible, structured, and annotated datasets that can be dynamically queried and analyzed [12], can be envisioned as companion infrastructure to support dynamic data access, processing and visualization of the growing data capital in research and healthcare. The digital biobank serves as a backbone structure for integrating diagnostic imaging, pathology, and NGS to allow a comprehensive approach. It should also be considered as a tool for the biomarkers discovery and validation in order to define multifactorial precision medicine systems supporting decision-making in the medical field [11]. A digital biobank needs an IT infrastructure equipped with a workstation, compliant operating system, RAM (8–16 GB), physical or cloud storage space (500 GB − 2 TB), stable internet connection (10–200 Mbps), office and security software functions [13].

On this premise, we would like to investigate procedures aiming at standardization and harmonization of data associated with diagnostic imaging, histopathology and NGS. This is directly linked to the potential data management in a digital biobank to allow a complete approach to diseases and also to make these data usable from artificial intelligence (AI) algorithms [14].

It is well-known that AI is becoming more and more important in data centric fields such as biomedical research and biobanking. However, the rapid development of AI technology is also accompanied by many ethical concerns and potential biases in AI algorithms when handling sensitive medical data, necessitating a careful balance between technological advancement and the ethical principles of patient privacy and fairness [15].

The study aims to address the following specific objectives:

- To review the current data standardization and harmonization initiatives, concerning all the domains and shared in common practice;
- To propose a comprehensive digital biobank (CDB) approach that integrates data from different domains. The approach is designed to address the identified standardization and harmonization needs, and could serve as a valuable tool for clinical decision-making in the field of precision medicine.

**Fig. 1:**
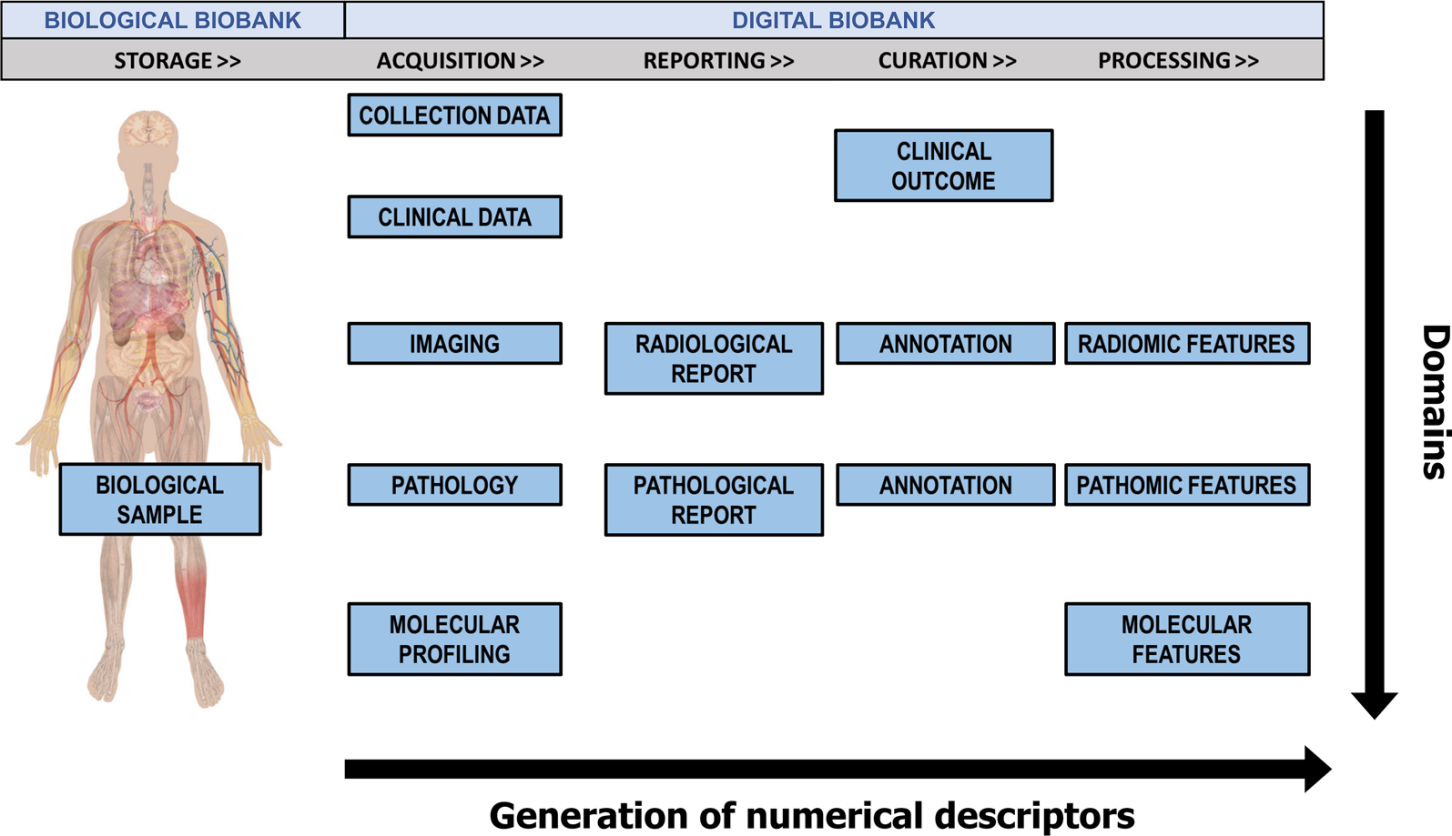
Overview of data included in a comprehensive digital biobank according to the generation of numerical descriptors (e.g. radiomic features extracted from radiological images [16], pathomic features from digital pathology images [17], as well as molecular features from molecular profiling [18]) during the sample lifecycle (horizontal increasing arrow) and to the integration of different domains (vertical descending arrow)

Figure 1 shows an overview of data types included in a CDB. Following the linear sequence for the generation of numerical descriptors, we represented (from the right side) all the data concerning storage and acquisition of a biological (e.g., tissue, blood, etc.) or digital (diagnostic or pathology image) sample together with the data provided by the reporting, curation, and processing procedures. On the other hand, following the vertical line, the integration of the diagnostic domain in each step of the sample lifecycle is achieved. The goal of a comprehensive approach should be the definition and application of specific standards for each kind of data and their integration across the domains. Various sources of information at different scales, e.g., clinical, imaging, pathology, molecular, and all the associated semantic, semi-quantitative and quantitative (of which -omics-) metrics, can support the clinical decision.

In the first section we will overview the concept of biobank, along with challenges related to both traditional and digital biobanks. In the second section we will focus on how a CDB could be implemented and standardized, including the identification of key use cases and scenarios addressing specific clinical questions. While always keeping in mind the necessary security and privacy regulations in accessing and retrieving biobank data [19, 20], we will tackle the missing aspects to envision workable solutions. Furthermore, our study aims to emphasize the role of biobanks as a tool for predictive research, interpreting them as a source of multifactorial information to set up predictive models supporting decision-making in the healthcare system. By evaluating the quality of quantitative features and using the reproducible procedures of the biobank, a standalone system can be established to generate predictive results starting from domain-specific data.

### Biobanks’ overview

From its first use in the scientific literature in 1996 [21], the term “biobank” has been defined in many different ways [22]. Nowadays, thanks to the efforts of biobanks networks, there is a consensus to define a biobank as a repository for the storage and retrieval of structured collections of human biological samples and associated data for present and future research use [23]. Data derived from different sources, such as bodily fluids, tissues, skin cells and other biological samples, through genomic and molecular processing, are associated with the medical records and potentially accessible by the researchers. With the growth of the biobanking field, many different types of data have been incorporated in these repositories, from the historical and annotated pathological information associated with the patient clinics to the new data coming from the advent of -omics science (genomics, transcriptomics, proteomics, metabolomics) [11]. International Organization for Standardization (ISO) standards and Standard Operating Procedures (SOPs) are integral aspects of the definition of a biobank.

The importance of standardization becomes evident in numerous challenges that arise when it is lacking in biobanking practices. For instance, without standardized protocols for sample collection and handling, variations in preservation methods and transportation procedures can compromise the quality of stored samples, impacting the reliability of subsequent analyses. In the absence of standardized data management procedures, maintaining accurate and comprehensive records becomes challenging, hindering the reproducibility of studies and traceability of sample characteristics. Ethical and legal compliance faces hurdles due to the absence of uniform guidelines, leading to uncertainties in obtaining informed consent and ensuring participant privacy. Quality control and assurance suffer when standard measures are lacking, risking issues such as contamination and mislabeling. The accessibility and sharing of samples are impeded without standardized practices, hindering global collaborations. Long-term storage conditions and data format standardization become critical for sample stability and integration of diverse datasets, respectively. In essence, adherence to ISO standards and SOPs is essential to address these standardization-related challenges by providing a framework for consistent practices and uphold the fundamental principles of sample quality, data integrity, and ethical conduct in biobanking. Standardization promotes transparency, reproducibility, and interoperability, ultimately enhancing the reliability and impact of biomedical research [24–26].

SOPs ensure the correct implementation of essential biobanking components (anonymization, samples’ acquisition, transport, preparation, analysis, storage conditions and terms of sharing). Regarding the use of SOPs, a series of technical specifications relating to pre-analytical procedures for human samples developed by ISO/TC 212 are available to biobanks. The final purpose is the standardization of pre and post analytical procedures, analytical performances, laboratory safety, reference systems and quality assurance. Unfortunately, although promoted by international networks, this process is still not homogeneous and adopted by all biobanks. Furthermore, it is necessary to share an internationally accepted and implemented ISO standard for biobanks, as the recently introduced ISO 20387:2018 [27]. It specifies the general competence, impartiality, and consistent functioning requirements for biobanks, including quality control requirements, to ensure biological materials and associated data of proper quality. This document applies to all organizations that conduct biobanking procedures for research and development purposes, including the management of biological samples for the study of circulating tumor markers. The global adoption of the ISO 20387:2018 standard for biobanking faces multifaceted challenges rooted in the diverse nature of biobanks and the substantial commitment required for accreditation. One key challenge involves the shift from the ISO 9001:2015 certification scheme to the more comprehensive ISO 20387:2018 accreditation, necessitating a transition from a focus on operational aspects to governance and management considerations. The extensive documentation and rigorous internal processes required for accreditation demand a significant investment of time, skilled resources, and financial commitment, ranging from EUR 15–25 K. Additionally, the varied sizes and capabilities of biobanks introduce challenges related to the implementation of standardized Quality Management Systems, infrastructure upgrades, and personnel training. Overcoming these challenges requires the development of flexible guidelines, financial support mechanisms for resource-limited biobanks, and a concerted effort to raise awareness globally. Strategies should also include fostering international collaboration, creating networks for the exchange of best practices, and emphasizing the long-term benefits of ISO 20387:2018 accreditation in terms of improved sample quality, stakeholder confidence, and research reproducibility. Overall, a carefully planned approach, involving stakeholders at different levels, is crucial for the successful and widespread adoption of the ISO 20387:2018 standard across the global biobanking community [27].

#### Beyond traditional biobanks

As described above, biobanks were originally intended as a collection of biological samples and associated clinical information. The progressive digitization process has made it possible to digitally archive an enormous number of images and several types of data. In 2014, the Imaging Biobanks Working Group (WG) of the Research Committee was established by the European Society of Radiology (ESR). It defined imaging biobanks as “organized databases of medical images, and associated imaging biomarkers, shared among multiple researchers, linked to other biorepositories” [28]. It is evident that an imaging biobank is not simply a system of archiving and transmitting images as are the PACS (Picture Archiving and Communication System) systems used in the hospitals. An imaging biobank not only allows the storage and retrieval of medical images and associated metadata, but the added value is that these data are linked to the imaging biomarkers, and to clinical, molecular, biological and genomic data. Imaging biomarkers are defined as characteristics extracted from the images of an individual that can be objectively measured and function as indicators of a normal biological process, a disease, or a response to a therapeutic intervention. Imaging biomarkers are complementary to conventional radiological readings to detect a specific disease or lesion, to quantify its biological situation, to evaluate its progression, to stratify phenotypic abnormalities and to assess the treatment response [29, 30]. This connection could be necessary for the researchers to find an association between phenotype and genotype [31, 32], to design and validate new imaging biomarkers, as well as to understand their biological significance, which may be a crucial point in precision medicine [33]. Another important difference is the organization of data and the way they can be retrieved. Whilst a PACS can be defined patient-based, in the sense that a query is based on the search for single-patient examinations, an imaging biobank can be defined population-based or disease-oriented as patients having a common disease are grouped and a user can query a specific study or disease [31]. The architectures for the creation of medical imaging and molecular imaging biobanks must incorporate advanced high-performance computing capabilities to allow high-throughput processing. Nowadays, institutions gather a whole spectrum of mostly digital information, including social, clinical, imaging and pathological records together with genomic profiles. Consequently, modern biobanking is shifting its focus from sample-driven to data-driven strategies. This implies that data management and integration has become a major component of contemporary biomedical research. In particular, the transition to digital biobanks enabled the amalgamation of diverse datasets, necessitating sophisticated data management and integration techniques. This shift has implications for the accessibility of data, requiring robust systems for efficient storage, retrieval, and analysis. Additionally, the move towards digital platforms facilitates collaborative research and accelerates advancements in precision medicine by enabling the exploration of associations between various data types [34]. In essence, the transition to digital biobanks underscores the pivotal role of data-centric approaches in shaping the landscape of biomedical research [35].

#### Biobanking networks and research infrastructures

In the initial stages of accreditation of a biobank, it is essential to be recognized by the own institution and to relate to the regional and national authorities responsible for managing public health. Biobanking networks aim to connect biobanks together to standardize institutional recognition procedures and coordinate the sharing of common strategies at European level. The biobanking field underwent a huge development with the fostering of such networks.

The Biobanking and BioMolecular resources Research Infrastructure (BBMRI)-European Research Infrastructure Consortium (ERIC) represents a reference as biobanking research infrastructure. The network was designed to operate across European countries with the aims of improving interoperability and giving quality management services to biobankers and researchers. Today the BBMRI-ERIC includes 20 countries and one international organization, making it one of the widest biobanks network [36].

Furthermore, the network has recently introduced a tool, the BBMRI Negotiator, to facilitate data sharing and collaboration among different biobanks, making data and materials rapidly and widely available to researchers. An online catalog has been established for the collection and presentation of data describing the majority of European biobanks [37].

During the state of emergency caused by the COVID-19 pandemic, BBMRI promoted the organized collection and safe sharing of COVID-19 patient samples, data and images. The collections were registered in a dedicated and publicly available directory.

Of note, the COVID-19 emergency has highlighted crucial lessons for future biobanking strategies [38, 39], particularly emphasizing the need for agile implementation tools alongside established standards. The recognition that the existence of standards doesn’t guarantee immediate applicability underscores the importance of developing tools that facilitate the swift adoption and interpretation of standards. Aiello et al. highlighted that, although the Digital Imaging and Communications in Medicine (DICOM) is the format used in the clinical acquisition routine, its limited adoption for the release of COVID-19 CT public datasets may indicate that the actual emergency conditions enhance the difficulty in finding suitable tools during emergencies, leading researchers to resort to alternative, more manageable formats [40]. Another important lesson is the imperative need for quality assurance, traceability, and financial investment in biobanking. The urgency of the pandemic has underscored the critical role of resilient infrastructure and well-trained personnel to ensure the safety and accuracy of procedures. Future strategies must prioritize these aspects to effectively handle emergencies [25].

Among the other existing infrastructures, the International Society for Biological and Environmental Repository (ISBER) is a global biobank organization that creates opportunities for networking, education and innovation. ISBER provides a forum for the dissemination of state-of-the-art policies, processes, and research findings; provides an international showcase for innovative technologies, products, and services; and promotes informal round table discussions where biobankers come together to connect and discuss hot topics in the biobanking industry sharing best practice [41]. Moreover, there are networks that bring together disease or pathology-oriented biobanks, such as for example EuroBioBank. It is a unique network of 25 rare disease biobanks located in 11 European countries. These biobanks store and distribute quality DNA, cell and tissue samples for scientists conducting research on rare diseases [42].

#### Imaging biobanks projects

A recent systematic review of existing image repositories shows that of the 54 selected biobanks containing images (of which 61.1% disease-oriented and 38.9% population-based) a relatively small proportion can be classified as imaging biobanks [43].

An example of one of the largest and most comprehensive worldwide biobanks is the UK biobank, a large-scale biomedical database and research resource, containing in-depth genetic, imaging studies and health information from half a million UK participants [44].

It is worth mentioning two registered European biobanks that have established organ and / or pathology-based images collections: the Central Biobanking facility at the Erasmus MC (Netherland) [45] and the BCU Imaging Biobank at Bio Check Up Srl (Italy) [46]. The former facilitates excellent scientific research using biomaterials (biological samples, images, clinical and epidemiological data), the latter is a non-profit biorepository aimed at the collection and storage of diagnostic images, derived descriptors and clinical data to foster scientific advances in imaging and biomarkers discovery. The Brain Images of Normal Subjects (BRAINS) Imagebank is designed to provide detailed structural brain imaging data of healthy individuals across the human life-course. The image bank, hosted by the Brain Research Imaging Centre at Edinburgh University (Scotland, UK), is a searchable database of integrated data sets already collected as part of research studies which include healthy (or control) subjects [47].

Several European projects aiming to build data infrastructure containing radiological images that are adequately cross-linked to corresponding -omics and health datasets. The euCanSHare project [48] was the first project designed to link these infrastructures for secure and integrated storage of heterogeneous data samples (incl. imaging, -omics, bio-samples and health data), with pilot validation for cardiovascular personalized medicine. Some European projects are under development as part of SC1-DT-TDS-05-2020 H2020 call “AI for Health Imaging’’: PRIMAGE (Predictive in silico multiscale analytics to support childhood cancer personalized evaluation empowered by imaging biomarkers [49]), CHAIMELEON (Accelerating the lab to market transition of AI tools for cancer management [50]), EUCANIMAGE (A European Cancer Image Platform Linked to Biological and Health Data for Next-Generation Artificial Intelligence and Precision Medicine in Oncology [51]), INCISIVE (A multimodal AI-based toolbox and an interoperable health imaging repository for the empowerment of imaging analysis related to the diagnosis, prediction and follow-up of cancer [52]) and ProCAncer-I (An AI Platform integrating imaging data and models, supporting precision care through prostate cancer’s continuum [53]). All these projects are devoted to testing and developing AI tools and analytics focused on the prevention, prediction and treatment of the most common forms of cancer while providing solutions to securely share health images across Europe.

Various approaches have been implemented by these projects, ranging from centrally collecting anonymized data to federated learning. For each project, specific harmonization methodology, ontologies and curation tools have been selected. The innovative aspects of the data infrastructures proposed by these projects are the compliance with FAIR (Findable, Accessible, Interoperable and Reusable) data principles, the General Data Protection Regulation (GDPR) and data quality standards, offering high computational performance, achieving interoperability, built for all involved stakeholders [54].

Two other European calls devoted to the development of cutting-edge services for the enhancement of research infrastructures funded two big projects: ISIDOR-e (Integrated services for infectious disease outbreak research [55]) and CanServe (Providing Cutting-Edge Cancer Research Services Across Europe [56]) support scientists and their research respectively in the field of infectious and oncological disease, providing free transnational access to a comprehensive portfolio of high-quality services, resources and expertise. The European funding initiatives underlined the importance being placed on testing and developing AI tools and analytics focused on preventing, predicting and treating the most common forms of cancer, while providing solutions to securely share knowledge of healthcare images across Europe.

#### Challenges

Given the above-mentioned evolution of biobanks, the associated challenges are also changing. In particular, the main challenges related to comprehensive biobanking can be sorted into three macro-groups related to standardization, reproducibility and integration.

A detailed description of each category is provided below, with next sections describing potential solutions.

1. **Standardization**: the first issue concerns the standardization of the formats for each domain (those vertically with respect to Fig. 1). Standardizing the formats for each domain is crucial for ensuring consistency and comparability of data across different sources. This includes not only the data itself, but also the methods used to acquire and store it. This can be challenging when dealing with domains that have not yet been fully standardized. However, standardizing the formats is necessary to ensure that the data can be easily shared, analyzed and compared, as well as made more suitable for creating predictive systems that support medical decision-making.
2. **Reproducibility**: To guarantee the reproducibility of the step towards the digital content (numerical descriptor) for each domain, it is necessary to follow clear and consistent data management procedures. This is crucial for both research purposes and healthcare services, as it allows results comparison, validation, replication, and dissemination. The basic assumption is that the user of the biobank should be able to expand the dataset starting from new biological samples or raw data (imaging or pathomics). Practically, a digital biobank should release, together with the raw and processed information, all the metadata useful for reproducing any procedure used to derive digital data from biological data.
3. **Integration**: Today, an important challenge of the biobanks is the development of imaging technological tools required for radiomic/pathomic analyses, integrating these features with genetic and clinical data in novel research approaches such as radiogenomics/radiopathomics. Features from different domains (last column of Fig. 1) should be put together in a single format to facilitate bioinformatics, multiomics, and multiassay. In this context, the biobanks’ contribution in integrating imaging and molecular data will represent an innovative approach to improve patients’ clinical management supporting the creation of predictive systems that could impact decision-making.

Each of these challenges cannot be treated ignoring regulatory and bioethical issues. Although the biobanking and the sharing of extensive databases could favor innovation and research in healthcare, they are considered potentially critical because of the accessibility to sensitive data. Regarding the protection of the patient and human biological material, a milestone is represented by the Declaration of Helsinki guidelines (1964). This document sets ethical principles including the importance of protecting the dignity, autonomy, privacy of the participants of research projects. To face this issue for biobanks, the World Medical Association (WMA) published the Declaration of Taipei to provide guidelines on the collection, storage, and use of identifiable data and biological material beyond the individual care of patients [57]. This declaration is the first international guideline to provide ethical directions about the complex issues that arise with activities associated with human databases and biobanks. Furthermore, the GDPR 2016/679 represents one of the most complete and shared tools worldwide for the protection of sensitive data and defines the techniques for pseudonymisation and anonymisation of personal data [58].

FAIR principles point out a path to follow, suggesting that the maximization of the utility of clinical/research data is obtained if these data are traceable, accessible, interoperable, reproducible, and of good quality, allowing study findings to be imparted and shared in a clear and understandable way. FAIR principles are not to be intended as a standard, but simply as a definition of good data stewardship practices [59]. The term `Findable’ implies data can be found online, typically through indexing in search engines. `Accessible’ means data can be retrieved directly or via an approval process. ‘Interoperable’ imposes data to follow standards. Finally, `Reusable’ requires the context of the data generation (metadata) is documented so it can be compared to or integrated with other data sets. These principles were initially developed for the academic world but have become an indispensable part of clinical research. Following these principles requires an application of standards to the various aspects of data collection and sharing. In relation to this aspect, Holub P et al. proposed FAIR-HEALTH principles [60], including additional components such as quality aspects related to research reproducibility and meaningful reuse of the data [61, 62]; incentives to stimulate effective enrichment of data sets and biological material collections and their reuse on all levels [60, 63]; privacy-respecting approaches for working with the human material and data. Ultimately, to overcome these challenges, digital biobanks must ensure that data and information are standardized, reproducible and integrated, following regulatory and bioethical guidelines [64]. Additionally, adhering to ISO standards and SOPs, as well as following the FAIR principles, will contribute to the success of the digital biobank model.

In the context of biobanks, the practical implementation of regulatory and ethical considerations, including GDPR and FAIR principles, involves a combination of ethical considerations, regulatory compliance, and technological measures to safeguard participant rights, ensure data quality, and facilitate responsible data sharing for research purposes. Therefore, it is vital for responsible and transparent management of human biological material and data [60, 65]. For example, BBMRI-ERIC research infrastructure focuses on providing access to these resources. Recognizing the legal limitations on “open access” due to privacy risks associated with large-scale sensitive human data sharing, the paradigm has shifted towards FAIR access principles in order to ensure that access is granted in a manner that respects privacy concerns and legal constraints. BBMRI-ERIC is also at the forefront of efforts to harmonize the application of GDPR to medical research through the development of a Code of Conduct on the European scale. These codes will provide specific guidelines for various domains, addressing the nuanced challenges posed by GDPR in the context of biobanking and promoting ethical and regulatory compliance [66].

Various initiatives have been launched to tackle the highlighted issues.

### Towards comprehensive biobanking

In this section, current biobanking initiatives will be critically analyzed considering the comprehensive biobanking perspective, identifying possible critical issues and solutions. Integration and digitalization efforts at various levels (standards, repositories, tools) will be examined.

#### Integration of standards

The standardization of procedures, file format and vocabularies is the first step to integrate heterogeneous data and to guarantee the functional and semantic interoperability. As will be emphasized in the following sections, each domain should have its own standard but in some specific domain, e.g., in radiomics, there is not yet a single standard universally approved (de facto) or imposed by the laws (de jure).

In fact, in the domain of radiomics, the lack of a universally approved standard poses several specific challenges to data integration efforts. The heterogeneity of imaging modalities, each with its unique file formats and data structures, complicates the standardization process. Diverse algorithms and software tools for feature extraction contribute to variations in feature definitions and extraction methodologies. Moreover, the absence of consensus on clinically relevant radiomic features hinders the development of standardized vocabularies. The interpretation of correlations between radiomic data and clinical outcomes varies among experts and institutions, adding to the complexity. The rapid evolution of imaging technologies and continuous advancements in analysis methods necessitate flexible standards that can adapt to technological progress. Interdisciplinary collaboration is crucial, requiring effective communication between radiologists, oncologists, data scientists, and other stakeholders. Additionally, addressing concerns related to data privacy, regulatory compliance, and the limited adoption of existing standards further underscores the need for a concerted effort to establish robust and widely accepted standards in the field of radiomics [67].

Therefore, a first significant effort is needed to bring together different standards to create a standard-based integration profile. This integration profile should aim to simultaneously describe clinical, imaging, biological, molecular and omics data, by defining the meaningful attributes to represent the data in each field in a standard way. This also requires an effort in the database design and in the establishment of how to organize and connect data so different from each other in a single structured repository.

The ESR started a collaboration with BBMRI-ERIC in 2014, recognizing the importance of integrating imaging and “omics” data. Therefore, this challenge is being addressed in the several European projects previously mentioned, which in fact aim to build data infrastructure containing radiological images cross-linked to corresponding -omics and health datasets. In particular, in the context of the PRIMAGE project, it has been proposed a first standard-based integration profile to link imaging data to biological sample data, typically included in a traditional biobank [49, 68]. To build this model of interoperability among heterogeneous data, the existing formats and ontologies for image and data description, the Minimum Information About Biobank Data Sharing (MIABIS) and DICOM, were considered as standard of reference. In this DICOM-MIABIS model, MIABIS has been expanded to the imaging field to also include image collections, and the expansion has been realized by adding to the MIABIS core a module based on the DICOM standard metadata. Since, as said, it is challenging to find a well-accepted radiomic standard, a special attention shall be paid to the description of the radiomic features extraction and the biomarker validation, which are fundamental data that add value to an imaging biobank. But certainly, this DICOM-MIABIS integration profile represents a first effort and a starting point for standardization of imaging data and metadata representation for data sharing. In a recent update, MIABIS Core 3.0 has been developed with 32 attributes describing Biobanks, Collections, Research Resources and Networks according to a modular structure that makes it easier to adhere to and to extend the terminology. Additional aggregate-level components have been prepared for imaging (DICOM-MIABIS) and for SOPs.

#### Integrated repositories

The critical issues related to the development of imaging biobanks often make it hard for a small research group to have its own biobank with a significant quantity of both radiomic and biological -omic data. A solution to this problem is the use of public databases containing multi-omic and imaging data with additional supporting data related to the images such as patient outcomes, treatment details, genomic, pathology and expert analyses [43].

Databases are available for different biomedical research fields with primary availability and development in the oncological and neurological fields. In the field of oncology, the US-based Cancer Imaging Archive (TCIA) [69] is a service that stores medical images of cancer patients in a large archive accessible for public download. DICOM is the primary file format used by TCIA for image storage, but TCIA does not enforce other standards for describing nonimage supporting data, such as treatment details and patient outcomes [70]. The connection of TCIA to the Cancer Genome Atlas [71] (TCGA-TCIA) represents the largest data repository in cancer research containing several primary sites and a large amount of available data (over 20,000 primary cancer and matched normal samples crossing 33 cancer types) [72]. Of note, some of the available information is not compliant with standards [40].

Imaging Data Commons (IDC) is a repository of publicly available cancer imaging data (radiology collections from TCIA and subsequently digital pathology images from Human Tumor Atlas Network (HTAN)), often linked with other types of cancer data, and co-located with cloud-based computational resources and big data analysis tools provided by the Google Cloud Platform [73]. The success of TCIA is measured by the number of scientific publications based on TCIA collections (2067 cumulative value until 2023), by the number of the browsed collections (207) and by the data usage. The NCI Cancer Research Data Commons (CRDC) is a cloud-based data science infrastructure that connects data sets with analytics tools to allow users to share, integrate, analyze, and visualize cancer research data to drive scientific discovery [74]. In the field of neurological and neurodegenerative as examples are available the National Institute of Mental Health (NIMH) Data Archive (NDA) [75] and the Laboratory of Neuro Imaging (LONI) [76]. Before the advent of open repositories, it was extremely difficult for an investigator to share and find datasets relevant to his research. The repositories allow researchers, engineers, educators to use their datasets collections to test and validate new hypotheses, to build new analysis tools and techniques, to show students interesting and specific use cases. In addition, a number of active research communities and collaborations have developed thanks to the sharing of specific multicentric collections. Some limitations include the lack of adequate descriptions of the collection, linkages to other databases, standard-compliant data formats, and the complete anonymization of metadata which leads to a loss of information fundamental to research. The main concern associated with using public databases is related to the risk of re-identification for individuals’ sensitive data. A key aspect is the application of data curation procedures and of robust de-identification techniques. Anonymization may no longer be appropriate, especially if individual-level data is to be shared. Some repositories apply access restrictions, but the decisional procedure and the data access criteria must be transparent. A further barrier to data sharing is the insufficient attribution of credits and the (mistaken) authors’ beliefs about ownership of data. Balancing the potential benefits of using public databases for research and healthcare advancements against the ethical and privacy concerns requires a delicate approach [77, 78].

#### Platforms for digital biobanking

From the technological point of view, a comprehensive biobank should have an optimized software architecture for the massive extraction of quantitative data and its association with other variables [79]. The main functional requirements are: (i) integration with current health information systems (i.e. DICOM sources, PACS, electronic medical records), (ii) modular extensibility in different components (.i.e. medical image visualization, database searching engines, back-end, front-end), (iii) scalability allowing for the wake up process of new storage units or servers, (iv) easy accessibility for clinical users and collaborators, (v) inference of AI models and data mining. The main challenges in the implementation of these technologies concern the security of protected health information, the lack of uniformity in metadata, the standardization of metadata, the vendor dependency and long-term sustainability. Historically, neuroimaging communities have been the most productive in developing platforms for the collection and management of DICOM diagnostic images. Noteworthy, the open-source software suite Extensible Neuroimaging Archive Toolkit (XNAT) was developed by the Neuroinformatics Research Group in St. Louis (Missouri, USA), to address and facilitate data management challenges in Neuroimaging studies. While XNAT supports mainly DICOM images and reports, it can, at least in principle, store data of different types [80]. XNAT relies on a three-tiered software architecture made of a PostgreSQL database back-end, a Java-based middleware tier usually deployed on an Apache Tomcat servlet container, and a web-based user interface. Specialized in the integration of neuroimaging data, the Collaborative Informatics and NeuroImaging Suites (COINS) is another platform that enables radiologists and researchers to easily manage questionnaires, neuropsychological, and clinical assessments and neuroimaging data (MRI, EEG, MEG, and genetic data) [81]. It was developed at the Mind Research Group (New Mexico, USA). COINS’s main strengths are the adoption of a centralized infrastructure and the well structured taxonomy for data and data sharing. At the time of writing, COINS supports only the DICOM format, but other data types could be zipped and uploaded via the web interface. The metadata schemas of XNAT and COINS are both structured using XML. One of the main limitations of XNAT is that the creation of a new data type (e.g., clinical variable or assessment) requires the construction of a new XML document and other operations that demand manual changes by an administrator with good informatic skills. On the contrary COINS allows a greater level of extensibility and the creation of customized clinical assessments to complement the neuroimaging scans. However, it does not support user-configurable fields for all the neuroimaging data types (only for MRI and MEG), and no explicit creation of new data types is available to integrate other data sources. Concerning the database, COINS stores all the metadata in an Entity-Attribute-Value (EAV) catalog, while XNAT adopts a mixed model using tables for the widely used data types along with an EAV representation for all the remaining metadata [82]. In terms of the scalability of the system, the EAV approach is less efficient in data retrieval and could affect the catalog performance. Both the repositories are equipped with a DICOM node to receive the imaging studies and a web portal for the users’ access.

## Comprehensive digital biobanking model

After the recognition of major challenges and criticisms in the clinical and research context, as well as of the integration and digitalization efforts, this section aims to define the key cases and scenarios of a comprehensive biobanking approach. We will account for the needs and requirements (related to standardization and harmonization) for the implementation of a CDB. A prerogative of the proposed approach will be “to invent as little as possible”, thus including current “standards”, when possible. On this premise, existing standards, procedures and initiatives will be introduced as pillars of the proposed comprehensive biobanking model, that will be discussed as a valuable tool for clinical decision-making in the field of precision medicine.

### Use cases and scenarios

The identification of use cases is key to highlighting the main challenges and criticisms in the clinical and research context and will help to steer the needs and requirements for the implementation of a comprehensive biobanking approach. First, it should be emphasized that a comprehensive biobank user (namely a researcher/clinician) has two different ways to interface with the digital biobank:

- **“Data Catalog” mode**: a Data Catalog is a collection of metadata, combined with data management and search tools, that helps analysts and other data users to find the data that they need, serves as an inventory of available data, and supplies information to evaluate the suitability of data for intended uses. According to this interface modality, the user does not access the digital biobank data, but can design a research project with the support of information derived from the catalog. Only after the approval of the project by the Ethical Committee, the data will be transferred to the user according to the Material and Data Transfer Agreement (MDTA);
- **“Data Access” mode**: thanks to this modality, the user can preliminarily consult the catalog, then he/she can access biobank’s collections with his/her account and thus perform the analyses directly on the platform. The details of which user has access to which data are typically managed on a project-by-project basis under the responsibility of the collection owners themselves.

It is worth noting that in the “Data Catalog” mode of interacting with a digital biobank, users are spared from immediate privacy concerns. Because this mode revolves around metadata and data management tools, researchers can design and assess research projects without accessing the actual biobank data. Since the focus is on  the information derived from the catalog rather than the data itself, there is no immediate need to inform subjects about the specific research being conducted. This is in stark contrast to the “Data Access” mode, where privacy issues come to the fore. In this mode, researchers need not only to consult the catalog, but also access the biobank’s collections for direct analysis. This requires informing patients about the use of their data, obtaining ethical approvals, and adhering to strict data access protocols, thus limiting the freedom to expose and analyze data without strict ethical considerations. Striking a balance between facilitating comprehensive research and ensuring patient privacy becomes imperative in the data access mode.

Concerning the possible use cases, these could be of two types:

- **Based on research questions**: a researcher/clinician needs a collection to address a specific clinical question about a pathology or population of interest. (e.g., can some radiomic features predict survival in a particular disease?).
- **Based on data integration and reproducibility**: a researcher/clinician needs external collection to augment his/her initial dataset, to reproduce results obtained by other researchers or to validate AI models.

**Fig. 2:**
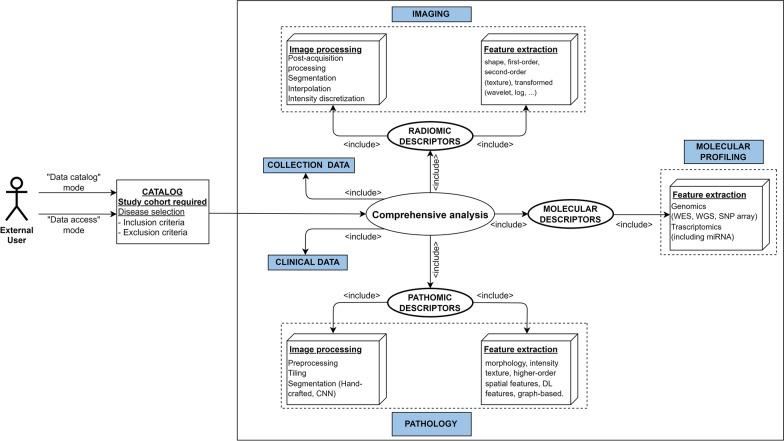
Use case diagram representing an external user (clinician/researcher) with the aim of performing a comprehensive analysis involving genomic, radiomic, and pathomic features of a specific tumor type or reproducing or integrating an already performed study. The figure also depicts the ways to interface with the digital biobank (e.g. “Data Catalog” or “Data Access” mode). *CNN* Convolutional Neural Networks; *DL* Deep- Learning; *WES* Whole Exome Sequencing; *WGS* Whole Genome Sequencing; *SNP*  Single Nucleotide Polymorphism; *miRNA*  MicroRNA.

Figure 2 shows both an example of a CDB that enables the implementation a study involving radiomic, pathomic, and genomic descriptors, and a use case where an external researcher or clinician that aims to explore a CDB based on specific criteria, for example to answer a research question. This figure is also representative of a use case where an external researcher aims to reproduce results obtained in another study. In this case all the above-mentioned challenges would come to the surface. To illustrate the outlined use cases, two real-world relevant scenarios can be considered, namely a multi-omic oncology study (e.g. radiogenomic or radiopathomic investigation), and, in view of the recently faced emergency, a study aiming at improving the accuracy and efficiency of COVID-19 diagnosis through AI-based segmentation. Concerning the multi-omic oncology study, a researcher may be interested in exploring the correlation between radiomic features extracted from radiological images and genomic markers [83](or pathomic features [84]) associated with clinical/pathological outcomes in a specific cancer type and could leverage the CDB to access the required material (e.g. raw images, molecular data, image annotations) to obtain numerical descriptors and/or explore pre-extracted features to reproduce previously obtained results. Moreover, the second real-world example involves a clinician or researcher who aims to develop an AI-based segmentation model for COVID-19 lesions to accurately identify and delineate lung alterations in COVID-19 patients [85]. Also in this case, the researcher can leverage the CDB to access a wide set of raw chest CT images and corresponding masks from COVID-19 patients, as well as to augment his/her initial dataset with other data contained in the CDB, to reproduce results previously obtained by other researchers or to validate the developed AI models.

### Standardization and harmonization initiatives

In this section, we will focus on solutions to implement the comprehensive biobank approach according to the requirements defined above. The prerogative will be to include standards, protocols, and standardization initiatives already existing and shared in common practice. A list of the current standards and standardization/harmonization initiatives related to all domains of Fig. 1 and functional for the development of a comprehensive digital biobanking approach is presented in Table 1. Table 1 includes standards and initiatives selected from an online semi-systematic review. In addition to the well-known DICOM and Health Level Seven (HL7) standards, standardization initiatives concerning storage of biological sample, data acquisition (collection and clinical data, imaging, pathology and molecular profiling), data reporting (radiology and pathology report), data curation (clinical outcomes, annotations) and processing (including both procedures and feature extraction) were selected. Only standardization or harmonization initiatives/alliances/communities that had a web reference, were recently updated, and provided a complete picture of the formats, data models, and operating procedures to be followed were selected. Exclusion criteria were therefore the partial information on the website, the absence of recent publications, the absence of clear protocols and guidelines. Taken together, these initiatives represent a huge opportunity to converge towards the interoperability of digital biobanks with clinical data management systems used in common practice, promoting the collection and sharing of real-world data, with a notable impact on the data quality and volume.

**Table 1:** Initiatives and standards functional to the development of a comprehensive biobank

Acronym	Extended name	Domain	Introduction date	Aim	Conformance standard (Y/N)	URL / references
MIABIS	Minimum Information About Biobank Data Sharing	Clinical	2012	Promoting a model of minimum information required to initiate collaborations between biobanks and enable the exchange of biological samples and data.	Yes (de facto)	https://www.bbmri-eric.eu/services/miabis/
HL7	Health Level 7	Electronic health information	1987	HL7 is a non-profit ANSI-accredited standards development organization that develops standards that provide for global health data interoperability.	Yes (consensus-based)	https://hl7.org
OMOP-CDM	Observational Medical Outcomes Partnership – Common Data Model	Health data	2008	Standardize the structure and content of observational data and to enable efficient analyses that can produce reliable evidence	No	https://ohdsi.github.io/CommonDataModel/index.html
CDISC	Clinical Data Interchange Standards Consortium	Clinical	1997	Standards to support the acquisition and submission and archive of clinical research data.	No	https://www.cdisc.org/standards
DICOM (SR, SEG, WG 26, CP-1705, CP-1764)	Digital Imaging and Communications in Medicine	Medical Imaging, Digital Pathology, Radiomics	1993	Enables the transmission, storing, retrieval, processing, and displaying of medical imaging information in a standardized format.	Yes (de jure)	[134]
IBSI	Image Biomarker Standardization Initiative	Radiomics	2016	Standardizing the extraction of image biomarkers from acquired imaging for the purpose of high-throughput quantitative image analysis.	No	https://ibsi.readthedocs.io/en/latest/ [92]
RQS	Radiomics Quality Score	Radiomics	2017	Checklist to ensure quality of radiomic studies.	No	https://www.radiomics.world/rqs [16]
OME	Open Microscopy Environment	Pathology	2012	Developing a data model for representation of image acquisition parameters, image experiment metadata, image analysis results.	No	https://www.openmicroscopy.org/ [146]
Feature DB		Radiomics/Patomics	2017	Building a comprehensive framework to support the generation, management and interrogation of large volumes of radiomic and pathomic feature sets.	No	[147]
MixS (MIGS, MIMS, MIMARKS, MISAG, MIMAG, MIUViG)	Minimum Information about (X) any Sequence (Genome, Metagenome, Marker gene, Single Amplified Genome, Metagenome-Assembled Genome, Uncultivated Virus Genome)	NGS	2005	Defining a set of core descriptors for genomes, metagenomes and the samples thereof.	Yes (de facto)	http://www.gensc.org/pages/standards-intro.html
MIAME, MINSEQE	Minimum Information About a (Microarray Experiment, a high-throughput sequencing experiment)	NGS	2001	Accelerating and supporting the effective sharing and reproducibility of functional genomics data.	Yes (de facto)	https://www.fged.org/projects/miame
QIBA EIBALL	Quantitative Imaging Biomarkers Alliance European Imaging Biomarker Alliance	Medical Imaging, Biomarkers	2007 2019	Standardizing methods to create biomarkers that meet a claimed performance (accurate and reproducible).	No	https://qibawiki.rsna.org/index.php/Main_Page https://www.myesr.org/research/eiball/

In addition, standards and standardization/harmonization will be analyzed and selected in light of well-known standards-related challenges, such as fragmentation of standards across institutions and countries or interoperability between different systems and platforms.

#### Clinical and regulatory data standardization initiatives

A critical issue is related to the standardization of clinical data to favor the sharing of medical information. Biological researchers and biobankers, being the producers and often the end-users of such data, have a pivotal role in enabling biological data integration. In this context, one of the most promising approaches is adopted by the BBMRI-ERIC infrastructure. The concept of MIABIS was introduced in 2012 by the Sweden BBMRI to facilitate sample collection and data sharing. Subsequently, it was further updated in 2016, upgrading the components defining biobanks, collections, and studies on an aggregated level [86]. The integrated data can then be used for retrieving data in queries in a structured and organized form. The MIABIS Core version 2.0 is currently used in different biobank registers and catalogs, that is, in the BBMRI-ERIC Directory; the development and the improvement of MIABIS is currently coordinated by the Common Service IT operations of BBMRI-ERIC [87]. Another proposal of sharing of clinical data in digital format are the interoperability standards (V2.x, V3, CDA, FHIR) promoted by HL7 International. HL7 is a not-for-profit, ANSI-accredited organization dedicated to providing a comprehensive framework and related standards for the exchange, integration, sharing and retrieval of electronic health information that supports clinical practice and the management, delivery and evaluation of health services. Another option is the Observational Medical Outcomes Partnership (OMOP) Common Data Model (CDM). It is an open community data standard, designed to standardize the structure and content of observational data and to enable efficient analyses that can produce reliable evidence. The CDM is designed to include all observational health data elements (experiences of the patient receiving health care) that are relevant for research use cases to support the generation of reliable scientific evidence about disease natural history, healthcare delivery, effects of medical interventions, the identification of demographic information, health care interventions and outcomes. Finally, an important initiative is supported by the Clinical Data Interchange Standards Consortium (CDISC), establishing standards to support the acquisition and submission and archive of clinical research data.

Taken together, these initiatives represent a huge opportunity to converge towards the interoperability of digital biobanks with clinical data management systems used in common practice, promoting the collection and sharing of real world data, with a notable impact on the data quality and volume.

#### Imaging standardization initiatives

The standardization of the medical imaging formats plays a crucial role in the effective use of the data and subsequent clinical decision-making. DICOM is the current *de jure* standard for the storage, retrieval and transmission of radiological and many other medical images, enabling the integration of multi-vendor medical imaging devices such as scanners, servers, workstations, printers, network hardware and PACS [40]. The DICOM file format contains mandatory and optional metadata describing the patient, examination details, and, in many cases, technical details of individual images (e.g., rows, columns, modality, manufacturer). The DICOM standard fully supports a series of key actions involved in the radiology workflow (de-identification, annotation, reporting), allowing to encapsulate in a single format much of the information necessary also for subsequent analytical phases. DICOM-Structured Reporting (SR) provides a versatile mechanism for communicating image-based measurements and supports both quantitative and qualitative evaluations using the TID 1500 template [88, 89]. A DICOM-Segmentation Object (SEG) is the standard way to encode segmentations defined as labeled image voxels [90]. For example, considering a typical radiomic workflow, the use of DICOM objects would allow an AI system to work with appropriately de-identified data, information related to the patient’s clinical status (DICOM-SR) and information on the localization of the region of interest (DICOM-SEG) within a single DICOM folder that can be useful to calculate the radiomic descriptors, thus favoring the aggregation of suitable data to develop reliable systems for classification or prediction of clinical outcomes.

While DICOM is widely used and well established, the Neuroimaging Informatics Technology Initiative (NifTI) format is also gaining recognition as a *de facto* neuroimaging file format, but it also has several other advantages that make it a popular choice in various imaging applications. Unlike DICOM, which primarily contains technical details of the images and is optimized for clinical use, NifTI provides a more straightforward and flexible way of storing and exchanging imaging data and its metadata, making it an attractive option for researchers and data scientists. One of its key benefits is its compatibility with a wide range of software platforms and applications, allowing for easy sharing of imaging data between different systems.

Harmonizing imaging data standards with other data types in the biobanking context poses significant challenges. The variety of data types collected by biobanks, including clinical, genomic, and imaging data, requires a comprehensive approach to standardization. Achieving interoperability between DICOM and NIfTI standards, as well as other formats, is complicated by the different storage systems and information models employed by different biobanks. Standardizing metadata across these disparate sources is crucial for seamless integration. Moreover, ethical and legal considerations, such as patient privacy and data sharing regulations, add layers of complexity to the harmonization process. Dealing with the large and complex nature of medical imaging data, along with ensuring both syntactic and semantic interoperability, further underscores the challenges. Ongoing updates to standards and the need for user training and adoption contribute to the multifaceted nature of harmonizing these standards in the biobanking landscape [28, 91].

Another important aspect concerns standardization and harmonization of numerical descriptors associated with diagnostic imaging (e.g., radiomics), as well as of procedures for obtaining these numerical descriptors. The lack of shared reference standards concerning data storage, the missing agreement on analysis procedures, and the feature reliability and reproducibility limitations affect radiomics. However, several initiatives have been launched to address these issues. Quantitative Imaging Biomarkers Alliance (QIBA) and European Imaging Biomarkers Alliance (EIBALL) initiatives include collaborating to identify needs, barriers and solutions to the creation of quantitative biomarkers, and accelerating the development of hardware and software to obtain accurate and reproducible quantitative biomarkers. In addition, the Image Biomarker Standardization Initiative (IBSI) is an independent international collaboration dedicated to standardizing radiomic analysis. In particular, the IBSI aims to address many challenges in 4 different specific areas: (1) standard nomenclature and common radiomic features, (2) radiomics image processing schemes, (3) data sets for validation and calibration, and (4) a set of reporting guidelines [92]. This group defined 174 radiomic features commonly used to quantify the morphologic characteristics and numerous others needed to define the quantitative information and tries to standardize the image processing steps of data conversion, post-acquisition processing, segmentation, interpolation, masking, and others. Such standardization is expected to make radiomics clinically useful and scalable for the integrated diagnosis service [93]. Limiting the radiomic analysis to the IBSI standardized features can facilitate the interchangeability of radiomic features across platforms [94].

Concerning procedures, a detailed reporting and documentation of radiomic studies is essential to develop this emerging field in terms of clinical translation and to improve the reproducibility of study outcomes. The Radiomics Quality Score (RQS) has been introduced to assess radiomic studies in terms of their compliance with best-practice procedures and to provide a reference guide for the drafting of manuscripts of radiomic studies [16].

#### Digital pathology standardization initiatives

Traditionally, pathologists assess and document features of traditional slides in diagnostic reports, which are then archived. With the introduction and advancement of digital pathology, the significance of slides has undergone a transformation, acquiring a “digital copy”, thus allowing for immediate reuse.

Digital pathology is a general term that refers to the process of digitizing histopathology, immunohistochemistry or cytology slides using whole-slide scanners, along with the interpretation, management, and analysis of these digitized whole-slide images (WSIs) using computational approaches (computational pathology) [95, 96]. The digital pathology slides can be stored in a centralized repository, enabling remote access for manual review by a pathologist or automated evaluation by a data algorithm.

Computational pathology uses advanced computational methods, either hand-crafted (pathomics [17]) or deep-learning-based, to extract valuable information from high-resolution WSIs that can be correlated with phenotypic features in different types of malignancies in association with the traditional histopathologic evaluation performed by pathologists [97]. Despite the growing demand, digital pathology is currently still limited due to several aspects [98]. First, the introduction of digital pathology in clinical practice is highly dependent on the standardization of procedures and file formats. The process of transforming glass slides into WSIs involves a series of phases: (i) pre-analytical (tissue procurement to fixation, processing, cutting, etc.), (ii) analytical (stain selection, validation) and digital (scanning, evaluation of monitor resolution, number of colors and distribution image format), (iii) post-analytical (analysis of results, the reporting of data, and machine learning application and sharing) [99]. Although there are still no robust standardization criteria, several encouraging initiatives have been proposed. The College of American Pathologists provided guidelines for the use of approaches involving digital pathology; moreover, other organizations such as the National Society for Histotechnology in the USA and the Royal College of Pathologists in Europe have initiated programs and recommendations for the implementation of digital pathology [100]. Based on the compelling need for data standardization and interoperability in digital pathology, there are ongoing efforts for the standardization of the representation and storage of pathology image data and analysis results [101, 102].

The DICOM Standard Committee WG-26 has put in a tremendous effort to support the use of DICOM in the pathology domain, and considerable progress has been made in incorporation of the information object model for pathology images, including WSIs. The use of DICOM for digital pathology images allows to achieve highly efficient pathology workflows and to easier manage WSIs together with images from other diagnostic domains [103–106]. While the DICOM standard has been extended to support digital pathology, it has seen little adoption in pathology practice. At the time of this writing, no  Food and Drug Association (FDA)-cleared digital pathology systems actually employ it natively [101]; however, at least one high-throughput WSI device (Leica Aperio GT 450 DX) outputs DICOM natively [107]. Generally, TIFF (Tagged Image File Format) or SVS (Aperio ScanScope Virtual Slide) file formats are preferred for various reasons. First, they can handle larger file sizes than DICOM thanks to lossy compression, which is important in digital pathology where WSIs can be several gigabytes in size. However, the compression potentially leads to degradation of image quality. Second, TIFF and SVS allow for faster access to images for review and analysis, without the need for decompression or conversion, and are more commonly used in open-source projects in digital pathology. Instead, DICOM addresses primarily IT experts who have the necessary technical expertise to implement it. This disconnect has resulted in an apparent lack of prioritization of interoperability, and vendors lack a compelling return on investment for building DICOM turn-key solutions. On the other side, DICOM provides a rich set of metadata, including patient information, image acquisition parameters, and annotations, while SVS and TIFF lack this level of information. Moreover, DICOM is a widely accepted standard in medical imaging, allows for seamless interoperability between different systems and platforms and provides long-term archiving, ensuring that images will be accessible and usable in the future, while SVS and TIFF may not be as well-suited for this purpose. As these standards are refined and implemented, we expect that open source and commercial software products will adopt these formats to enable interoperability across different imaging and software systems.

Another important aspect concerns the standardization and harmonization of pathomics workflow. The challenges stem from several factors, including the heterogeneous nature of the data, the variability in image acquisition and processing, and the lack of consensus on the best methods for obtaining the numerical descriptors. One challenge is to ensure the comparability of results obtained from different imaging modalities, such as bright-field, fluorescence, and electron microscopy. Another challenge is to ensure the reproducibility of results, as the variability in image acquisition and processing can lead to different results even when the same image is analyzed multiple times. To address these, it is necessary to establish consensus-based standards and guidelines for image acquisition, processing, and analysis, as well as for the generation of numerical descriptors that ensure consistency and reproducibility.

Despite the previous considerations, the integration of digital pathology data in a digital biobank could represent a groundbreaking advancement, especially when compared to the more established digitalization in radiology. While radiology has been at the forefront of the digital revolution in medical imaging, the digitalization of pathology images is a more recent development that holds immense promise, especially in bridging the gap across multiple scales (e.g., molecular, microscopic, macroscopic) in the study of diseases, particularly in oncology, where understanding the molecular and cellular intricacies of tumors is critical for accurate diagnosis, prognosis, and treatment decisions [108].

#### Next generation sequencing standardization initiatives

The possibility of managing NGS data confers the opportunities to adopt a personalized approach to the patient. Despite the efforts of these international projects to encourage sharing in processing, analyses, and output of genomics data, there is not yet a single shared direction relating to the management of NGS data. Indeed, the actual proposed standardized procedures and data formats, as well as comprehensive quality management considerations, are not yet fully followed. There are many initiatives promoted and aimed at standardizing genomics data in the fields of (i) reporting standards initiatives, (ii) data analysis and quality metrics projects, (iii) file format, data analysis and quality control tools, and (iv) data integration initiatives. Concerning (i), the basic approach to better exchange and integration of data contributed by different laboratories using different sequencing technologies is the adoption of MIGS-MIMS (Minimum Information about a Metagenomic Sequence). MIGS represents a minimum information checklist that is aimed at standardizing the description of a genomic sequence maintained by the Genomic Standards Consortium; indeed, this organization has also developed an extension of MIGS to support metagenomic data sets called MIMS [109, 110]. For this aim, the Genomic Data Commons Data Portal requires one to provide a specific set of metadata, to contribute to the platform [111]. Other international initiatives such as MIAME (Minimum information about a microarray experiment), MINSEQE (Minimum information about a high-throughput sequencing experiment) are adopted to facilitate the workflow of genomic data standardization [112]. They are proposed by the Functional Genomics Data Society (FGED) and define a minimum set of metadata for high-throughput sequencing to ensure quality, documentation, and reproducibility of experiments and sharing of data. Concerning (ii), the FDA – National Center for Toxicological Research has underlined the necessity of comparability between results obtained from different platforms. The MicroArray Quality Control Project (MAQC) is a project addressed to the reliability and reproducibility of cross-platform gene expression analysis as well as the development of standards and quality guidelines [113, 114]. Other similar international projects focused on quality metrics efforts were the Critical Assessment of Microarray Data Analysis (CAMDA) and the Normalization and Transformation Ontology (NTO).

About (iii), there are several data standards that have become *de facto*, meaning they are widely accepted and used without being officially sanctioned. These standards cover a range of topics including sequences, variants, and experiments. For sequences, the FASTQ format is widely used to store and exchange DNA and RNA sequence data along with their associated quality scores. This format is the starting point for most genomic analysis and has become a cornerstone of many genomic analysis pipelines, as the quality scores are critical for the assessment of the quality and reliability of the sequence data [115]. Another important data format in genomics is BAM (Binary Alignment Map) /SAM (Sequence Alignment Map), which is used for storing and sharing the results if aligning the sequences in FASTQ to a reference genome. BAM, the binary version of SAM, is a compact and efficient format for storing substantial amounts of alignment data, while SAM is a human-readable format. BAM/SAM files are commonly used for large-scale genomic analysis and can be used for tasks such as read visualization, quality control, and downstream analysis. There are several types of quantitative data that can be generated after processing BAM and SAM files, including sequence alignments, quality scores, coverage, structural variations, epigenetic analysis, genomic variants [116]. Concerning the latter, the VCF (Variant Call Format) format is widely used for storing and sharing information about genomic variants, such as single nucleotide polymorphisms (SNP) and insertions/deletions. VCF files provide a standardized way of describing and comparing genomic variants across different samples and are widely used for genomic data exchange and storage [117].

It is not necessary to convert BAM/SAM files into VCF files in all cases, as BAM/SAM files contain information about the alignment of the sequences and VCF files contain information about genomic variants. Depending on the specific analysis tasks, either format may be more appropriate, or both formats may be used in conjunction. For example, if the goal is to perform variant calling, the BAM/SAM file would first be used to align the sequences and the resulting alignment would then be used as input for a variant caller to generate a VCF file [117].

Regarding (iv), many standard initiatives and efforts have been described and are available for the different datasets; they are continuously proliferating but unfortunately not necessarily in harmonizing ways. An important initiative, focusing mainly on the standardization of genomic data is carried out by Global Alliance for Genomics and Health (GAGH), an organization that could help to develop the interoperability to unlock the great potential of genomic data [118]. To further accelerate the standardization process, several international organizations took part in the creation of physical standards for omics data. The US National Institute of Standards and Technology (NIST) has focused on the standardization of sample preparation through a number of projects. An ongoing project is the Genome in a Bottle (GIAB) consortium [119] focused on adapting procedures established for whole-genome sequencing to the clinical environment [120]. Other initiatives are promoted by the Association of Biomolecular Resource Facilities (ABRF), a network focusing on standardization and optimization with the objective to develop guidelines; in detail, the ABRF-NGS group aims to identify the optimal methods and strategies for NGS projects, comparing the performances of different NGS platforms [121, 122].

It will be essential for biobanks to follow these international standardization initiatives to make the different types of stored data accessible, manageable, and reusable, promoting an upcoming application of NGS data in clinical practice.

It is worth noting that the implementation of genomic standards within the biobanking framework poses several practical challenges, particularly in the context of data sharing and privacy. This complexity stems from several remarkable features that make genomic data different from other health data, such as the direct relationship between genomic-associated information and prognosis, as well as the presence of significant commonality among blood relatives individuals. Genomic data, with its stability and identification potential, raises privacy concerns that challenge conventional health data privacy models. Advances in privacy technologies are improving genomic data sharing, but regulatory and ethical guidelines need further enhancement. Addressing these challenges is crucial for empowering individuals to actively contribute to scientific research, advancing genomic data sharing and benefiting medical research [123, 124].

### Proposed approach

**Fig. 3:**
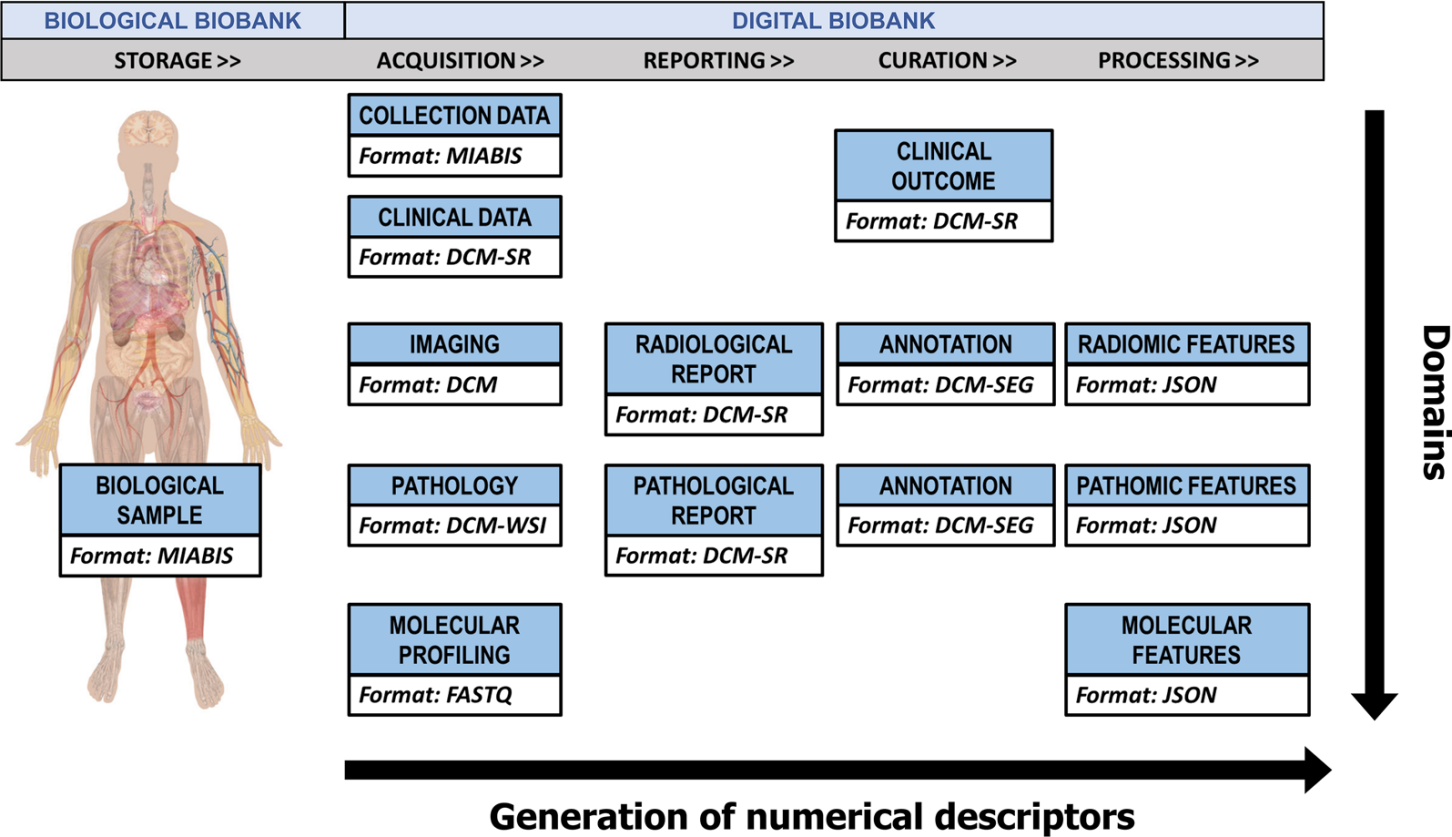
Proposed integrative approach accounting for standardization/harmonization of each diagnostic domain and integration among multidisciplinary domains, together with the harmonization/standardization concerning the generation of numerical descriptors associated with each single domain. The approach involved the use of MIABIS, DICOM and FASTQ as they are established standards in the common practice to describe raw and derived data from clinical imaging, pathology, and next-generation sequencing domains. JSON format was proposed to store and interchange domain-specific numerical descriptors. *MIABIS* Minimum Information About BIobank data Sharing; *DCM (DICOM)* Digital Imaging and Communications in Medicine; *WSI* Whole Slide Imaging; *SR* Structured Report; *SEG* Segmentation; *JSON* JavaScript Object Notation

Based on some of the existing initiatives identified in the previous sections, we proposed a comprehensive biobanking approach that could address the identified standardization, reproducibility, and integration needs. The proposed approach is based on the use of standards and data formats already existing and shared in common practice (Table 1). Looking at Fig. 3, the challenge is to develop an aggregate database model that is functional for the integration among multidisciplinary domains (vertical view) and the generation of numerical descriptors associated with each domain (horizontal view). According to our proposed model (Fig. 3), the storage and the acquisition of the biological samples will be managed using the MIABIS standard. Instead, the key actions of the imaging workflow will be supported by the DICOM standard, which allows to encapsulate in a single format much of the information necessary for the subsequent analytical phases both for radiological and pathological domains [40]. Indeed, the proposed approach is based on the use of DICOM for the acquisition of both radiologic images and WSI images. On this line, DICOM-SR is proposed for the encoding of clinical data, radiology and pathology reports, as well as clinical outcomes. Furthermore, all image annotations (both associated to radiology and pathology) will be encoded in DICOM format using DICOM-SEG. These choices are also motivated by the existence of a theoretical model of DICOM-MIABIS integration [68].

Of note, despite the obvious advantages of DICOM in terms of with respect to interoperability and enterprise integration, challenges concerning the DICOM extension for WSI are still present and include for example the storage and transmission strain for large datasets, necessitating ongoing standardization for size-related limitations; the global vendor adoption; the choice of image compression methods and limited support in existing archives. Collaborative efforts and ongoing standardization are crucial for overcoming these challenges and realizing the full potential of DICOM in digital pathology [101].

In the molecular domain, we have proposed the use of FASTQ as the format for molecular profiling [125]. This choice is based on several advantages offered by the FASTQ format. FASTQ is a widely used and well-established format in the field of genomics and NGS data, and it provides reliable data storage, ease of use, and compatibility with existing NGS data analysis pipelines. Additionally, the FASTQ format includes quality scores for each base, which can be used for error correction and quality control during data analysis. By using FASTQ for molecular profiling, we aim to ensure the integrity and accuracy of the molecular data stored in the biobank.

It should be considered that, while leveraging FASTQ for molecular profiling in a digital biobank brings numerous advantages, integrating this data with imaging and pathology data introduces may require developing custom interfaces or middleware to ensure seamless data interaction. This is because FASTQ files have a unique format tailored for molecular data, that is completely different from the imaging formats. In fact, FASTQ data typically operates at a granular level, dealing with individual nucleotides. Integrating this high-resolution molecular information with imaging data, which may be volumetric and multi-dimensional, poses challenges in scaling and correlating information accurately. In addition, integrating these results with imaging or pathology-derived features may introduce analytical complexities, requiring sophisticated computational approaches, robust infrastructures, potentially impacting storage costs and retrieval times, and expertise in both genomics and imaging informatics.

Concerning the data processing and the generation of numerical descriptors (namely radiomic, pathomic and molecular features features), we propose to use JSON (JavaScript Object Notation) format [126]. JSON is a lightweight data format that makes it easy to transfer and store huge amounts of data. This makes it ideal for use in a digital biobank. Moreover, JSON is formatted in a human-readable way, and it is supported by the most common programming languages and tools, allowing for easy and portable data management. JSON data can also be hierarchically structured and easily parsed by computer programs, making it easier to organize and manage data in a digital biobank and to automate data processing and analysis tasks [126]. This is directly linked with the possibility of integrating JSON data with machine learning algorithms, allowing for the creation of complex and personalized prediction models that can handle large amounts of data [82].

DICOM is trialing the use of JSON for encoding the output of AI algorithms (e.g., risk prediction of skin disease) in DICOM-SR format. The goal of this trial is to harmonize with the machine learning community where JSON is the preferred format for algorithm output. Support for the JSON format has been added to the DICOM Standard in Part 18 as the DICOM JSON Model [127]. This model describes how different DICOM value representations can be encoded in JSON and allows for seamless integration of JSON data with DICOM objects.

Based on these considerations, JSON emerges as a powerful tool for structuring and organizing data within a digital biobank in several scenarios, thereby enhancing the efficiency, reproducibility, and compatibility of data processing and analysis tasks across different modalities and research objectives. For example, one can imagine a scenario where the biobank contains diverse datasets, including DICOM images from radiological studies, molecular data in FASTQ format, and associated clinical metadata. JSON can be employed to create a unified data structure that encapsulates information from these different modalities. The hierarchical nature of JSON allows for the representation of patient details, experimental parameters, and imaging annotations in a single, coherent format. This simplifies data retrieval and analysis by providing a standardized structure that machine learning algorithms can easily interpret and process.

In the context of radiomic analyses, JSON can be employed to store extracted features from DICOM images [128]. Each JSON object could represent a specific radiological study, encapsulating details such as image metadata, segmentation information, and a structured array of radiomic features. This format ensures that radiomic data are stored in a coherent and easily accessible manner, facilitating subsequent analyses, comparisons, and the application of machine learning algorithms.

We also promoted the use of IBSI-compliant software for radiomic feature extraction, such as PyRadiomics [128], that can also be used starting from DICOM input images with the file name pointing to a DICOM-SEG, thus automatically obtaining radiomic features without any intermediate steps. This allows for a reproducible feature extraction that can be achieved under real clinical conditions that usually involve DICOM objects. Of note, PyRadiomics supports the use of JSON as a format for storing quantitative data [128]. This compatibility allows for easy storage and analysis of radiomic features in a digital biobank and supports the use of JSON as a format for quantitative data in medical imaging and radiomics.

Overall, the promotion of IBSI-compliant software for radiomic feature extraction marks a forward-thinking approach that significantly strengthens the reproducibility and reliability of radiomic analyses. Adherence to the standards set by the IBSI ensures a consistent and uniform framework for defining and calculating radiomic features. This adherence eliminates variability in feature extraction methods across different software tools and platforms, providing a common language for the field. The standardized definitions not only enhance the transparency of radiomic analyses, but also enable seamless interoperability, allowing researchers to achieve comparable results and facilitating cross-study comparisons. The adoption of IBSI-compliant software within the research community contributes to a more standardized and reliable landscape in radiomics, addressing critical challenges in the pursuit of meaningful and reproducible insights from medical imaging data [129].

As these standards are refined and implemented, it is expected that open source and commercial software products will adopt these formats as their default data models for image analysis results to enable interoperability across different imaging and software systems to facilitate easier development and integration of new data management capabilities.

Efforts should also be made to address the challenges related with the missing harmonization of ontologies between standards associated with multidisciplinary domains (the DICOM-MIABIS model constitutes a first example of effort towards this direction [68]), and the limited support for standards in existing healthcare data platforms, that could be addressed by integrating platforms or creating a platform with interconnected modules. The benefits of improved interoperability and data consistency make these efforts valuable for advancing the impact of biobanking in research and healthcare.

## Discussion

Based on the huge amount of heterogeneous information belonging to different diagnostic domains, there is an urgent need to make this information available and suitable to promote scientific research and technological development [3]. The management of such cross-domain heterogeneity has always been an open challenge, especially within the intricate framework of precision medicine. The essence of precision medicine lies in tailoring treatments to the unique profiles of individual patients, which increases the complexity of handling diverse datasets. This challenge is particularly acute in oncology, where the lack of a universal therapeutic approach is acutely recognized. Tumor heterogeneity between patients and even within the same patient over time complicates the establishment of standardized treatments [130]. The effective organization of biomedical data in biobanking infrastructures is crucial to enable a comprehensive approach to clinical studies as well as the development of AI tools supporting clinical decision-making [131, 132]. The main challenges are related to the (i) standardization, (ii) reproducibility and (iii) integration of data and procedures. In this study, we propose a comprehensive digital biobanking approach that could address the identified standardization and harmonization needs and serve as a valuable tool for clinical decision-making in the field of precision medicine. Of note, we harnessed digital biobanks as a source of multifactorial information containing standardized and curated imaging data along with clinical, molecular, and pathological data.

First, we introduced the concept of traditional and digital biobank, reporting some of the most well-known biobanking research networks and projects, and reviewed the state-of-the-art of the harmonization initiatives in the field of biobanking (i.e., standards, repositories, platforms). Moving from traditional to digital biobanking presents several challenges, especially concerning infrastructure requirements and data security. Upgrading from traditional storage methods to digital systems requires significant and expensive infrastructure changes. Continuous monitoring, updates, and migration to newer storage technologies are essential to preserve data integrity. Sensitive information requires strong encryption methods, access controls, and continuous monitoring to prevent breaches or unauthorized access. Digital biobanking raises ethical concerns regarding the secondary use of stored biological samples and associated data. Addressing these challenges requires collaboration among experts in biobanking, data management, cybersecurity, and regulatory compliance.

Furthermore, digital biobanks need to ensure that data and information are standardized, reproducible and integrated, following regulatory and bioethical guidelines and adhering to ISO standards and SOPs, as well as considering that the stewardship and management of scientific data needs to adhere to the FAIR principles [133]. This will contribute to the success of the digital biobank model to achieve its full potential as a scientific resource.

On this basis, we have proposed an integrative standardization/harmonization approach that encompassed (wherever possible) the use of standards and procedures that are already used in common practice. From the point of view of the domains, we chose to focus on the most advanced diagnostic domains (primarily in the field of oncology), namely clinical imaging, pathology, and NGS. We considered both the integration of these domains, as well as the generation of numerical descriptors associated with each single domain through robust data curation and data processing procedures. Our approach involved the use of MIABIS, DICOM, and FASTQ as they are established standards in the common practice to describe raw and derived data from the chosen domains. MIABIS was used to store and exchange data elements that describe the collection of biological samples [87]. DICOM was established for the acquisition, reporting, and curation (annotation and clinical outcomes definition) of both radiological [134] and WSI images [106]. Although the DICOM standard is designed to incorporate specific characteristics of the patient and the examination characteristics in the metadata, much of the information needed for molecular imaging analysis is not included.

FASTQ was used for storing NGS-generated raw data [125]. It is worth noting that, although several types of genomic analyses (e.g., array comparative genomic hybridization [135]) are used depending on the scientific question and available resourced, we structured our proposal based on a typical NGS pipeline due to its massive use for large-scale, high-resolution ultra-high-throughput, scalable, and fast genomic analyses [116].

Since the JSON format natively suits the hierarchical format of DICOM metadata, we proposed to use the JSON format to store and interchange domain-specific numerical descriptors. Furthermore, it is the preferred format for encoding the output of AI algorithms, is widely supported by major programming languages, and can be linked to formal ontologies [136].

To the best of our knowledge, this is the first study that foresees new models of standardization and integration among multidisciplinary domains, also proposing robust data curation and processing pipelines to obtain reproducible numerical descriptors associated with each diagnostic domain. In fact, dealing with standardized and harmonized procedures and data not only provides a conducive environment for advancing -omics studies (e.g., radiomics, pathomics, genomics), but also offers opportunities to explore the potential links between quantitative -omics data and clinical outcomes of patients with specific diseases [3, 18, 84]. In this regard, also Lu et al. [18] underscore in their review the significance of integrating various -omics modalities, including pathomics, radiomics, and genomics, to advance prognostic assays and delve into the potential links between quantitative -omics data and clinical outcomes in patients with specific diseases. Of note, they not only emphasize the importance of integration, but also highlight the challenges, potential opportunities, and avenues for future works.

Another noteworthy study by Izzo et al. [82]. focused on addressing the challenge of growing metadata heterogeneity in the biomedical field developing digital repositories with flexible and extensible data models, as in the case of modern integrated biobanks management. In particular, they proposed a novel flexible and extensible JSON-based data model to describe heterogeneous data in a generic biomedical scenario. They first describe how to incorporate the model inside the XTENS (eXTensible Environment for NeuroScience) digital repository to support heterogeneous data management in a generic biomedical science scenario. Then, they tested the model focusing on a specific use case of an integrated biobanking management, where different information (clinical, histopathological, genomic…) could be queried, integrated, and shown in a structured view. The JSON metadata schema they proposed aimed at describing and integrating in a highly flexible but consistent format heterogeneous datasets and information in biomedical science, both for clinical and research support, with a focus on biobanking and multidisciplinary biomedical research.

Overall, the implementation of the proposed comprehensive digital biobanking approach offers a range of advantages for clinicians, researchers, and patients alike. Firstly, the digital biobank ensures a complete and centralized repository of study information for research purposes. By standardizing and integrating medical images, molecular profiles, and patient data, the biobank improves data management, ensures interoperability between different systems, and makes it easier to access, exchange, and analyze large amounts of data. Notably, the integration of clinical outcomes, radiological, and digital pathology annotations, as well as the use of radiomic, pathomic, and molecular features, allows for the development of predictive models for various diseases, improving diagnosis and treatment planning.

It is important to understand the compliance of the standards with FAIR principles; this would be fundamental to accomplish the requirements of clinical research in terms of data sharing and management. As highlighted by Aiello et al. for the DICOM standard, further efforts are needed by researchers, clinicians, and companies to promote and facilitate the use of standards to increase the value of imaging data, according to FAIR [40]. In addition, implementation of the FAIR principles within the numerical descriptors’ fields (e.g. radiomics) can facilitate its faster clinical translation [137].

Therefore, in the proposed CDB approach, adherence to FAIR principles is paramount for effective data management. This entails not only ensuring that all incorporated standards are FAIR-compliant but also implementing FAIR principles to address numerical descriptors, thereby maximizing the utility of clinical and research data, emphasizing traceability, accessibility, interoperability, reproducibility, reusability, and data quality. Importantly, the notion of “reusability” extends to both human and machine utilization. Consequently, the proposed approach prioritizes making data machine-readable to harness the full potential of modern technologies. Within the realm of numerical descriptors, the Radiomic Ontology project offers a Python library for FAIR radiomics analysis, serving as a valuable resource to facilitate the seamless transition of research efforts into clinical practice [138].

It is worth noting the importance of including numerical descriptors within digital biobanks not only for what concerns the improvement of diagnostic-molecular knowledge and the direct implications on decision-making (e.g., in oncology where the personalized treatment approach is pivotal), but also for what concerns the regulatory and economic point of view. At the regulatory level, the proposed approach promotes and is directly applicable to federated solutions, where the raw data can remain in the proprietary site, exposing only the derived numeric descriptors [139]. The federated approach not only adheres to regulatory requirements but also facilitates scalability by allowing each site to manage and control its raw data. This decentralized structure contributes to the sustainability of the model, as it aligns with data protection norms and supports long-term collaborative efforts.

From an economic point of view, the advantages of sharing digital information are undeniable, just consider the enormous difference in costs between the conservation of biological samples and digital data and the limitation of aliquots of the biological sample compared to the unlimited possibility of reproducing digital data [11]. This economic efficiency holds significant implications for the scalability and sustainability of the biobanking model. However, as highlighted in this work, all these advantages require particular care in the management of digital data.

The centralized storage of large amounts of patient data in a digital biobank provides researchers with access to a rich resource for medical research, enabling the development of new treatments for various diseases. By reducing the need for repeated tests and imaging, and by improving patient outcomes, the implementation of a digital biobank can also help to reduce healthcare costs in the long term.

Storing data in standardized formats and using structured reports helps to ensure the accuracy and consistency of the data, resulting in improved data quality. Digital biobanks typically employ robust security measures, such as encryption and access controls, to protect sensitive patient data, ensuring privacy and confidentiality. They are designed to oversee enormous amounts of data and can be easily scaled to accommodate growth in the number of patients and types of data being collected. This results in time efficiency, as healthcare providers and researchers can save time and increase efficiency, leading to improved patient care and faster progress in medical research.

It is also crucial to emphasize that the effort towards the implementation of procedures aiming at standardization and harmonization of data associated with diagnostic imaging, histopathology and NGS is fundamental also to make these data usable by AI algorithms for predicting clinical outcomes.

Of note, the work by Kondylakis et al. [140] delves into the transformative potential of AI in the realm of medical imaging, emphasizing the need for extensive and harmonized datasets in AI development, particularly for cancer-related medical imaging. The collaborative efforts of five EU projects aim to create ethically compliant, quality-controlled, and GDPR-compliant big data infrastructures. These platforms seek to seamlessly integrate large-scale data with AI algorithms, establishing sustainable AI cloud-based systems dedicated to developing trustworthy and reliable models in cancer care. The study’s key points include the challenges of data access, the development of a common data model, and the importance of a European Union meta-tool repository to streamline efforts and minimize duplication in a dynamic field such as medical imaging.

A completely different field of medical AI is connected to clinical reports, which clinicians typically tend to make like a narrative text. AI can help to structure or extract specific text parts from routine pathology reports for further scientific purposes [141]. All the reports could be better used for any scientific purpose if it would be easier to search for different disease entities, and this could increase the value of millions of biospecimens and images which are currently stored in biobanks.

However, although out of the scope of our work, it should also be mentioned that the pathway for regulatory approval is a key roadblock in the clinical adoption of AI-based prognostic and predictive tools. One of the principles for regulatory permission includes the necessary explanation of how the software works and their translation to clinical practice (AI-based techniques are perceived as being a black-box and lacking interpretability) [140, 142].

The trasformative impact of AI on biobanks encompasses streamlining processes, improving data accessibility, and synergizing with biobanking to revolutionize cancer research and enhance patient-centric healthcare strategies. AI-powered predictive modeling can also aid in the strategic prioritization of research initiatives within biobanks. By analyzing historical data and identifying patterns, AI algorithms can assist researchers in identifying the most promising datasets for further investigation. This predictive capability not only optimizes research efforts but also contributes to resource efficiency by directing attention towards areas with higher potential for significant scientific advancements and contribution to a deeper understanding of diseases [14].

Among the issues emerging from our work, it should be considered that, besides the diversity of data, there is also a wide variety in the models built for homogenizing and storing them. This is the reason we opted for not including ontologies in our semi-systematic search for standardization initiatives. In fact, ontologies are usually developed for describing limited sets of data and cannot scale when other types of data need to be stored using the same model. More than this, various expert groups are performing extensions to ontologies that are not synchronized and compatible with each other, thus leading to several variations of the same ontology, which complicates the ontology selection and its reuse. In addition, ontologies have language-dependency [143].

Collaboration among expert groups to establish synchronized and compatible ontology extensions could enhance their usability and reduce variations. Alternatively, the exploration of domain-agnostic data representation frameworks may provide a solution to explore [144].

Moreover, we did not consider the domain of biomedical signals (EEG, ECG, …), which, although not yet associated with -omics domains, it deserves attention since it can allow link physiological information to other diagnostic parameters [145]. Future research should thus extend its scope to incorporate biomedical signal domains, exploring standardized approaches for their integration into comprehensive data models.

Another topic of discussion emerging from our work concerns the type of data to be included in digital biobanks. Although the topic is open and debated, we have an example that we could follow, consisting of the BCU Imaging Biobank [46]. This biobank is digital only and approved as a biobank by BBMRI. Therefore, although there is always a link to biological repositories in the definition of a digital biobank, we can also assume the absence of a biological sample. Along these lines, it can be said that the digital biobank would remain a biobank since it inherits from the traditional biobank all the procedures for certifying its content, including, for example, all issues related to the rights and ownership of collections.

In addition, since the aim of our work was limited to frame possible functional solutions for the implementation of a digital biobank, trying to solve the issues arising from standardization, reproducibility, and integration, the development of an IT infrastructure for data sharing was outside the scope of the work.

In conclusion, this work has proposed a CDB model that could improve the management, standardization and sharing of data in compliance with ethical norms.In particular, examining the current state of the art, the need emerged to build, starting from the current reference standards, an integrative framework that can guarantee effective exploitation of the full potential of complete digital biobanks. This issue has been addressed by proposing a standardization model of numerical descriptors that can be derived from each specific diagnostic domain. Ultimately, this work shows that with further standardization efforts it is possible to implement digital biobanks that represent the driving force to promote the development of data-driven and multi-domain tools that can facilitate the effective implementation of precision medicine.

## Data Availability

Not applicable.

## Abbreviations

AI: Artificial Intelligence
ABRF: Association of Biomolecular Resource Facilities
BBMRI-ERIC: Biobanking and BioMolecular resources Research Infrastructure-European Research Infrastructure Consortium
BRAINS: Brain Images of Normal Subjects
CAMDA: Critical Assessment of Microarray Data Analysis
CDB: Comprehensive Digital Biobank
CDISC: Clinical Data Interchange Standards Consortium
COINS: Collaborative Informatics and NeuroImaging Suites
CRDC: Cancer Research Data Commons
DICOM: Digital Imaging and Communications in Medicine
EIBALL: European Imaging Biomarkers Alliance
ESR: European Society of Radiology
FAIR: Findable, Accessible, Interoperable and Reusable
FDA: Food and Drug Association
FGED: Functional Genomics Data Society
GAGH: Global Alliance for Genomics and Health
GIAB: Genome in a Bottle
GDPR: General Data Protection Regulation
HL7: Health Level 7
HTAN: Human Tumor Atlas Network
IBSI: Image Biomarker Standardization Initiative
IDC: Imaging Data Commons
ISBER: International Society for Biological and Environmental Repository
ISO: International Organization for Standardization
JSON: JavaScript Object Notation
LONI: Laboratory of Neuro Imaging
MAQC: MicroArray Quality Control Project
MIABIS: Minimum Information About BIobank Data Sharing
MIAME: Minimum Information About a Microarray Experiment
MIGS: Minimum Information About a Genome Sequence
MIMS: Minimum Information About a Metagenomic Sequence
MINSEQE: Minimum Information About a high throughput SEQuencing experiment
MDTA: Material and Data Transfer Agreement
NDA: National Institute of Mental Health (NIMH) Data Archive
NGS: Next-Generation Sequencing
NIfTI: Neuroimaging Informatics Technology Initiative
NIST: National Institute of Standards and Technology
NTO: Normalization and Transformation Ontology
OMOP-CDM: Observational Medical Outcomes Partnership-Common Data Model
PACS: Picture Archiving and Communication System
QIBA: Quantitative Imaging Biomarkers Alliance
RQS: Radiomics Quality Score
SNP: Single Nucleotide Polymorphisms
SOP: Standard Operating Procedure
SR: Structured Reporting
SVS: Aperio ScanScope Virtual Slide
TCIA: The Cancer Imaging Archive
TCGA: the Cancer Genome Atlas
TIFF: Tagged Image File Format
VCF: Variant Call Format
WG: Working Group
WMA: World Medical Association
WSI: Whole-Slide Image
XNAT: Extensible Neuroimaging Archive Toolkit
XTENS: eXTensible Environment for NeuroScience
